# Quality improvement in the time of coronavirus disease 2019 – A change strategy well suited to pandemic response

**DOI:** 10.1017/cem.2020.386

**Published:** 2020-05-01

**Authors:** Shawn Mondoux, Jennifer Thull-Freedman, Shawn Dowling, Katie Gardner, Ahmed Taher, Rakesh Gupta, Sachin Trivedi, Heather Lindsay, Annie Finlayson, Simon Berthelot, Edmund Kwok, Lucas Chartier

**Affiliations:** * Division of Emergency Medicine, Department of Medicine, McMaster University, Hamilton, ON; † Department of Pediatrics, University of Calgary, Calgary, AB; ‡ Department of Emergency Medicine, University of Calgary, Calgary, AB; § Department of Emergency Medicine, Dalhousie University; ¶ Division of Emergency Medicine, Department of Medicine, University of Toronto, Toronto, ON; # Department of Emergency Medicine, University of Saskatchewan, Saskatoon, SK; ‖ Department of Emergency Medicine, University of British Columbia, Vancouver, BC; ** Department of Emergency Medicine, University of Manitoba, Winnipeg, MB; †† Department of Family Medicine and Emergency Medicine, Université de Laval, Quebec City, QC; ‡‡ Department of Emergency Medicine, University of Ottawa, Ottawa, ON

**Keywords:** Administration, patient safety, quality improvement

Quality improvement (QI) is a science that is built upon testing small changes and quickly learning from the results observed. Changes in normal operations, either as a deliberate experiment or externally imposed, have the ability to teach us about our systems, our teams, and ourselves. In many ways, we are further away from “normal” operations than we have been in a long while. Despite the declaration of an international pandemic^1^ and the reality that we, as emergency department (ED) providers, are the frontline in this fight, it seems that many Canadian EDs are experiencing a quiescent period that may not have been afforded to our colleagues in other countries. ED volumes are down across the country, yet we are no less busy, as we are readying ourselves for what has already been a defining healthcare event of our time. At a moment where evidence and guidelines are limited, we should look to QI to provide us with useful strategies in terms of preparation, operationalization, and delivery of healthcare.

In order to create system change, QI often incorporates change management strategies to influence a group of individuals to adopt new behaviours. In Kotter's classic framework on organizational change, this concept is the first step: “creating a sense of urgency.”^2^ The time and resources typically used to communicate the need for change are spared. ED teams aren't asking themselves IF they should change; they are asking HOW they should change (and how to do it in the most expedient way possible). The medical treatment of coronavirus disease 2019 (COVID-19) is unlikely to be the greatest challenge facing ED providers; rather, the greatest challenge is likely to be the implementation of complex new care processes.

As we enter this new reality together, we have also suddenly found ourselves in an information-rich yet knowledge-poor environment. Departments around the world are sharing their protocols for managing patient surges, conducting protected intubations, and preserving ventilator capacity and personal protective equipment (PPE). Social media, #medtwitter and local chat groups have made the dissemination of information seamless across time zones and international borders. These often are helpful, yet can also create local anxiety and fear, especially in the absence of good medical evidence. The protocols that are functional at other sites may be more harmful than beneficial locally in the absence of robust testing driven by the tenets of QI.

**Figure:**
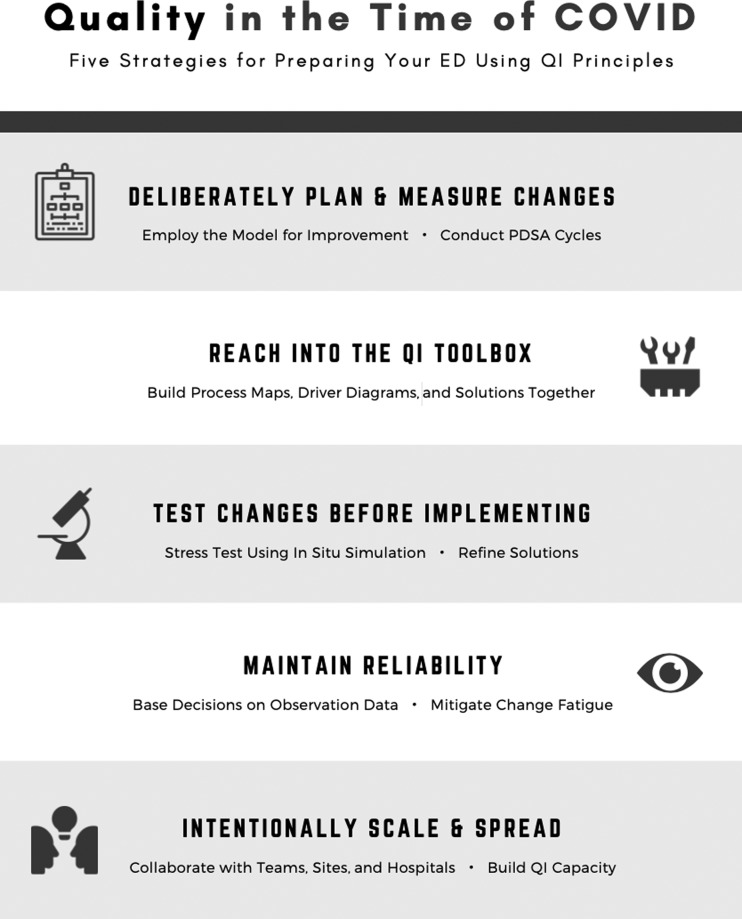

QI methods are uniquely suited to the rapid testing of new processes. Carefully selected measures allow assessment of the impact of changes in the real-world environment. In doing so, we are able to identify processes that, despite being effective elsewhere, are not useful or require adaptation locally to add value. When clinical trials are not feasible during tests of change, the importance of making decisions based on observation is paramount. The practice of watching work where it takes place is referred to in Lean terms as “going to the Gemba.”^3,4^ Learning from observable tests of change incorporates local intangibles such as team composition, performance, and anxiety, and it allows us to make decisions based on outcomes. This process is agnostic to expert opinion, popular vote, social media following, or the loudest voice in the room.

The following is a pragmatic list of how QI may add value to the ED in the midst of this pandemic:

1. **Process changes must have a deliberate planning and measurement strategy, based on established improvement tools.**
  The need to adapt quickly in the absence of clear evidence makes the use of established improvement models particularly important. The Model for Improvement is foundational in planning an effective system change.^5^ The model begins by clarifying the specific need for improvement and identifying how improvement will be determined. It proceeds with identifying specific changes to be tested and then testing these changes in the planned experimentation format of Plan-Do-Study-Act (PDSA) cycles.^6,7^
2. **Reach into the QI toolbox.**
  QI science provides useful tools and strategies for achieving change. While our current situation may lead us to feel that there is no time for use of formal strategies, use of a few key tools may allow us to achieve our desired results more efficiently. Consider tools such as driver diagrams, process map, and flowcharts as enablers of change.^8^ These concepts can help a team quickly prepare a complex workspace for new demands. During this pandemic, our colleagues, with a variety of expertise, specialist knowledge, and drive to contribute should be given space to propose solutions and develop prototypes to solve departmental problems. These skills can be beneficial for inviting and incorporating the collective ideas of a team.
3. **Test changes before implementing.**
  A common change pitfall is to implement before testing. In situ simulation can be a highly effective method for initial tests of change. For example, protected intubation and protected code blue protocols can be sourced from peer hospitals and refined through frequent iteration. Areas of emphasis may include donning and doffing PPE safely, communicating from within negative-pressure rooms, respiratory arrests in low-acuity areas, and system-wide simulations that include emergency medical services and intensive care unit services. These allow for systems to identify latent safety threats to both staff and patients before they happen to active decompensating COVID-19 patients.
  Small tests of change with a single patient can help determine the viability of a concept. This is often what is tested in the simulation environment. Despite this, the question of broad implementation of these concepts requires scaled testing or PDSA cycles.^6,7^ These repetitive tests should be done with differing teams, in multiple environments, and often with several simulations happening simultaneously to model advanced pandemic scenarios and identify the largest number of latent safety threats. This type of implementation stress-testing should be considered for all new initiatives because they may reveal unintended consequences.
4. **Maintain reliability.**
  Planning and leadership decisions can and should be augmented with data on what is actually happening on the frontlines rather than assumptions or opinions. However, the typical ways in which data are collected, aggregated, and presented in “normal” times may not be efficient enough for the required pace of change in a pandemic. As such, a simple observation of work in progress can be highly beneficial in understanding threats to reliability. For example, knowledge about errors in PPE use could be based on a few hours of dedicated observation in the ED by a redeployed staff. An assessment of adherence to doffing best practices could be based on the observations of an independent observer relative to an established checklist, rather than depending upon participation in education or the presence of reminders in the environment.
  Acknowledge that change fatigue will occur and take steps to mitigate. Frontline clinicians will have difficulty finding signals in the noise of the current information-saturated environment. We need to accept that this will continue to occur over the course of the pandemic. Accepting the challenges of the protocols to our end-users and making the right thing to do the easy thing to do is of utmost importance. As an example, some sites have elected to post a protected-intubation checklist on the door to each ED resuscitation room. Standardization and simplification, in process and communications, follow the tenets of the hierarchy of effectiveness.^9^
5. **Intentionally scale and spread.**
  Rogers’ classic Diffusion Theory^10^ described spread of innovation as dissemination over time through a social system. The opportunity to communicate and spread innovation has increased exponentially in recent years with social media technologies. Collaboration between teams, sites, and hospitals may be a great method to decrease “investment costs” at the early stages of planning and development. Most hospitals are deploying local human resources to develop the same protocols, responses, and strategies to solve common problems. The best use of our resources may be collaborative design followed by local refinement using QI testing principles. Furthermore, processes that are standardized across larger geographical areas often gather more public and administrative support. When successful changes are ready for dissemination, models such as the Institute for Health Improvement's Framework for Spread may provide strategic guidance.
  The scale and spread concept may also be applied to the development of QI capacity. Many departments and leaders have made use of distributed leadership as part of their pandemic response. Providing these distributed leaders with knowledge and skills in QI may enhance their effectiveness and simultaneously build QI capacity for future challenges. This strategy is most effective if there is a robust central coordination of efforts.

The science of QI was built for times like the current COVID-19 pandemic: a rapidly evolving situation that requires a deliberate and methodical approach, sensitive to local context, focusing on pragmatic adaptations while ensuring that we don't forget the ripple effects of all of the changes. As improvers, as leaders, and as clinicians, we must go forward and improve our environment in the safest and most effective ways possible. We must empower others to do the same, while supporting them to do it rigorously. It is evident to all in the healthcare system that COVID-19 has brought a new world. The principles of QI will help us navigate it successfully.
